# Exosomal miRNA-155 and miRNA-146a are promising prognostic biomarkers of the severity of hemorrhagic fever with renal syndrome

**DOI:** 10.1016/j.ncrna.2022.10.003

**Published:** 2022-10-27

**Authors:** Irina Gilyazova, Elizaveta Ivanova, Valentin Pavlov, Guzel Khasanova, Aliya Khasanova, Adel Izmailov, Dilara Asadullina, Gulshat Gilyazova, Guoqing Wang, Ilgiz Gareev, Ozal Beylerli, Elza Khusnutdinova

**Affiliations:** a Institute of Biochemistry and Genetics, Ufa Federal Research Center of the Russian Academy of Sciences, 450054, Ufa, Russia; b Bashkir State Medical University, 450008, Ufa, Russia; c Department of Pathogenobiology, The Key Laboratory of Zoonosis, Chinese Ministry of Education, College of Basic Medicine, Jilin University, Changchun, Jilin, China; d Republican Clinical Oncological Dispensary, Ufa, 450054, Republic of Bashkortostan, Russia; e Republican Clinical Infectious Diseases Hospital, Ufa, 450015, Republic of Bashkortostan, Russia

**Keywords:** miRNA, Target genes, Hemorrhagic fever with renal syndrome, Biomarker, Prognosis

## Abstract

**Introduction:**

Hemorrhagic fever with renal syndrome (HFRS), caused by Orthohantaviruses, occupies one of the leading places among natural focal human diseases, for which there are no modern accurate and highly sensitive diagnostic methods. To improve this situation, a better understanding of the Hantavirus pathogenesis of HFRS is required. Determination of the expression level of exosomal microRNAs (miRNAs) in the serum/plasma of patients makes them potential biomarkers for diagnosing and predicting HFRS. The purpose of this study was to analyze the expression level of miRNA-146a, miRNA-126, miRNA-218, miRNA-410, miRNA-503 and miRNA-155 in patients with HFRS at different stages (fever stage, polyuric stage and convalescence stage) and with different severity of the course this disease

**Materials and methods:**

The moderate group of patients with HFRS included 105 patients, the severe group included 99 patients, and the severe group with complications included 84 patients. Blood samples from patients with HFRS for molecular genetic analysis were collected three times: during the initial febrile period (days 1–4 of illness), the polyuric period (days 15–22 of the disease), and during convalescence. Total RNA isolation was performed using the exoRNeasy Midi Kit (Qiagen, Germany). Quantitative real-time PCR (qRT-PCR) was performed using the miRCURY LNA SYBR Green PCR Kit (Qiagen, Germany) and the LightCycler96 real-time PCR product detection system (Roche, Switzerland).

**Results:**

When comparing the expression level of exosomal miRNAs in groups of patients with different severity of the disease, a statistically significant increase in the expression level of miRNA-146a was revealed in patients with severe HFRS with complications (Fold change 2.694; p = 0.0022) compared to the group with a moderate disease form, as well as an increase in miRNA-155 expression in patients with severe and severe HFRS with complications compared to the moderate form (Fold change 1.861; p = 0.0492; Fold change 1.976; p = 0.001, respectively). Comparative analysis of the expression level of other miRNAs in patients with HFRS at various stages and with different severity of HFRS did not reveal any statistically significant results (P > 0.05).

**Conclusions:**

MiRNA-155 and miRNA-146a may be promising prognostic biomarkers in HFRS. However, further investigations are needed to evaluate the changes in the expression of miRNAs and the network of genes that can be potential targets for the studied miRNAs in order to elucidate the molecular mechanisms that can influence the occurrence and development of HFRS.

## 1 Introduction

Orthohantaviruses (Hantaviridae) are enveloped RNA viruses that belong to the most widespread zoonotic viruses transmitted by rodents [1]. These viruses are the etiological agents of two clinical forms of human disease: hemorrhagic fever with renal syndrome (HFRS) in Eurasia and Hantavirus cardiopulmonary syndrome in America. Every year from 150,000 to 200,000 cases of orthohantavirus diseases are registered in the world, with most cases of HFRS occurring in Asia, mainly in China [2]. In Russian Federation, HFRS occupies a leading position among all natural focal human diseases. In the Russian Far East, the annual incidence of HFRS is caused by two orthohantaviruses: Hantaan (genetic variants widespread in the Far East and Amur region) and Seoul [3]. Puumala virus (PUUV) is the main Hantavirus that causes HFRS in Europe. The number of patients diagnosed with HFRS in Europe is increasing and amounts to more than 3000 cases per year [1]. Almost 90% of all cases of HFRS infection registered in Russian Federation occur in the Volga-Ural region (VUR). Particularly high rates were noted in the Republic of Bashkortostan - this is the largest focus of HFRS in the VUR [3].

Symptomatic PUUV infection leads to a mild form of HFRS known as epidemic nephropathy (NE). The hallmark of NE is proteinuria followed by acute kidney injury (AKI), in some cases requiring hemodialysis. The most common symptoms include fever, general malaise, myalgia, gastrointestinal discomfort, blurred vision, and acute oliguria. At the site of outbreaks, most hospitalized patients present with mild to moderate to severe clinical symptoms, and in particular no fatalities. It is believed that the pathogenesis of PUUV is mainly due to increased vascular permeability, thrombocytopenia, and increased immune response due to infection of vascular endothelial cells (ECs) in various organs of the human body [4].

Despite numerous attempts to study this disease, the pathogenesis of viral hemorrhagic fevers remains largely unknown. It is believed that the course and outcome of the disease depends on the viral load, the genetic profile of patients and the immune response [2]. Active research is currently underway to fill gaps in knowledge about the pathogenesis and diagnosis of HFRS. Until now, there are no test systems for predicting the course of the disease that would have high accuracy, sensitivity, and specificity.

Promising markers in this respect may be microRNAs (miRNAs) - endogenously expressed RNA molecules 18–22 nucleotides long, which suppress gene expression at the post-transcriptional level by binding to the 3′-untranslated region (3′-UTR) of target mRNAs and play a significant role in various biological processes, including cell cycle, apoptosis, cell proliferation and differentiation. Although the role of miRNAs in various viral infections has been actively studied in recent years, there are only a few publications devoted to the study of the role of miRNAs in Hantaan viral infection. A number of miRNAs have been identified that play an important role in the control of the barrier function of ECs and may be involved in the pathogenesis of HFRS [[5], [6], [7], [8], [9], [10]].

MiRNAs released from virus target cells is reported to regulate viral infection and replication, and viral factors in turn modulate intracellular miRNA expression. It is assumed that the virus affects key miRNA-regulated reactions occurring in endothelium cells, responsible for maintaining vascular integrity, thereby causing inflammation and disruption of the integrity of the vascular barrier. The aim of this study is to analyze the expression level of exosomal miRNA-146a, miRNA-126, miRNA-218, miRNA-410, miRNA-503 and miRNA-155 in plasma in HFRS patients at different stages of the disease.

## 2 Materials and methods

### 2.1 Patients

For all HFRS patients the diagnosis was established by highly qualified doctors after collecting clinical and anamnestic data and obtaining the results of laboratory and instrumental examinations. All patients and healthy volunteers were examined at the Republican Clinical Infectious Diseases Hospital (Ufa, Russia). The use of human samples was approved by the Ethics Committee of Ufa Federal Research Center of the Russian Academy of Sciences (Ufa, Russia) and Republican Clinical Infectious Diseases Hospital (Ufa, Russia). Written informed consent was obtained from each participant prior to enrollment. The diagnosis of HFRS was confirmed by the method of fluorescent-antibody (MFA) test with a diagnosticum of the corresponding serotype. The diagnostic criterion was a fourfold or more increase in antibody titer in the second serum sample taken at intervals of 7–10 days. ELISA-HFRS-Puumala-IgM reagent immunoassay was used to detect class M antibodies to Puumala Hantavirus serotype (FGUP “NPO Microgen” of Russian Federation Public Health Ministry). Inclusion and exclusion criteria are presented in Table 1.

**Table 1:** Inclusion and exclusion criteria.

Inclusion criteria	Exclusion criteria
Age 18–50 years	Under 18 years old and over 50 years old
Voluntary consent of the patient to participate in the study	Withdrawal of the written voluntary consent of the patient to participate in the study
Duration of illness less than 5 days	Smoking and presence of other infectious diseases
The presence of a confirmed diagnosis of HFRS (with an increase in the titer of specific antibodies in paired sera by 4 or more times)	Individuals with no serological confirmation of the diagnosis of HFRS and mild forms of the disease
No comorbidities	
•Kidney disease	
•Diseases of the cardiovascular system,	
•Endocrine pathology	
•Tumors	
•Other infectious diseases	
•Autoimmune diseases	

**Note:** HFRS, hemorrhagic fever with renal syndrome.

All patients were divided into 3 groups depending on the severity of the disease course- a group of patients with a moderate course (n = 105), a severe course (n = 99), as well as a group of patients with a severe course and complications (n = 84). Separately, a group of healthy individuals (n = 100) who had never had HFRS and did not have chronic kidney disease was collected. The assessment of the severity of the condition of patients with HFRS was carried out in accordance with the classification of Sirotin [11]. At the same time, a complex of clinical and laboratory parameters was taken into account, consisting of the duration and severity of fever, manifestations of general toxic symptoms, renal syndrome (the level and duration of oliguria and/or anuria, azotemia), hemorrhagic syndrome, the degree of hemodynamic disorders and complications (toxic shock syndrome (TSS), disseminated intravascular coagulation (DIC) and AKI). In patients with severe HFRS, hemorrhagic syndrome was manifested by hemorrhages in the sclera, in the subcutaneous tissue at injection sites, nasal and gastrointestinal bleeding; in urine tests - macro - and microhematuria. When establishing the period of the disease, Sirotin's classification was also used, according to which 4 periods are distinguished: initial or febrile (1–4 days of illness), oliguric (5–9 days of illness), polyuric (from 9 to 15–22 days of illness) and convalescence (after 22 days of illness). The clinical characteristics of the examined are shown in Table 2.

**Table 2:** Clinical characteristics of the examined patients.

Symptoms	Forms of the disease					
	Moderate		Severe		Severe with complications	
	abs. n = 105	%	abs. n = 99	%	abs. n = 84	%
Fever	105	100	99	100	84	100
Headache	105	100	99	100	84	100
Lethargy	9	8.5	48	48.5	58	69
Meningeal symptoms	0	0	32	32.3	28	33.3
Hyperemia of the face and neck	89	84.7	37	37.4	28	33.3
Paleness of the skin	6	5.7	60	60.6	84	100
Аcrocyanosis	0	0	0	0	39	46.4
Scleral vascular injection	93	88.6	99	100	84	100
Visual impairment	38	36.2	74	74.7	73	86.9
Nausea	45	42.8	76	76.8	73	86.9
Vomit	33	31.4	63	63.6	73	86.9
Bradycardia	72	68.6	44	44.4	0	0
Tachycardia	11	10.5	55	55.5	84	100
Dyspnea	0	0	55	55.5	73	86.9
Backache	90	85.7	99	100	84	100
Abdominal pain	33	31.4	90	90.9	84	100
Decrease in diuresis	88	83.8	99	100	84	100
Liquid stool without pathological impurities	18	17.1	54	54.5	44	52.4

### 2.2 Laboratory and instrumental methods

Laboratory and instrumental methods performed on patients included a general blood and urine test, a biochemical blood test to determine the level of urea, creatinine, aminotransferases, a urine test according to Nechiporenko, Zimnitsky, a hemostasiogram (blood clotting time, prothrombin time, activated partial thromboplastin time (APTT), thrombin time, and fibrinogen), renal ultrasound, and electrocardiography. If necessary, patients underwent chest x-ray, blood tests for electrolytes (K+, Na+, Ca2+) and other studies. Daily blood pressure, pulse rate and body temperature were measured, as well as the amount of fluid received per day, and daily diuresis.

### 2.3 Blood samples collection and exosome isolation

Blood samples from patients with HFRS for molecular genetic analysis were collected three times: during the initial febrile period (days 1–4 of illness), the polyuric period (days 15–22 of illness), and during convalescence. Thus, each group included three samples from each patient with HFRS at different stages of the disease (in the course of treatment). Patients' blood was taken into special vacutainers with RNA stabilizers. Exosomes were isolated from blood plasma obtained by double centrifugation at 4 °C (10 min at 1900 g and 15 min at 3000 g), followed by isolation of total RNA. The rest of the plasma was immediately frozen and stored at −80 °C.

### 2.4 Isolation of exosomal RNA and reverse transcription (RT)-PCR

Exosomal RNA was isolated from 1 ml of filtered blood plasma using the exoRNeasy Midi Kit (Qiagen, Germany) according to the manufacturer's protocol as described previously [12].

The reaction mixture for reverse transcription contained 2 μL of total RNA, 8 μL of the main mixture containing reverse transcriptase as part of a 10x miRCURY RT Enzyme mix (Qiagen, Germany), 5x miRCURY SYBR Green RT Reaction Buffer (Qiagen, Germany), exogenous controls for isolation, reverse transcription, and amplification, and RNase free water (Qiagen, Germany). The reverse transcription reaction was carried out under the following conditions: 42 °C - 60 min, 95 °C - 5 min and 4°C-∞. The sequence of all primers used in the present study is provided in Table 3.

**Table 3:** Sequence of all primers.

miRNA/Ref. gene	Primer Sequence (5′-3′)
miRNA-146a	RT: GTCGTATCCAGTGCAGGGTCCGAGGTATTCGCACTGGATACGACAACCCA
	Forward: GCCGCTGAGAACTGAATTCCA
	Reverse: GTGCAGGGTCCGAGGT
miRNA-126	RT: GTCGTATCCAGTGCAGGGTCCGAGGTATTCGCACTGGATACGACGCATTA
	Forward: GCGGCGGTCGTACCGTGAGTAA
	Reverse: CAGTGCAGGGTCCGAGGTATT
miRNA-410	RT: GTCGTATCCAGTGCAGGGTCCGAGGTATTCGCACTGGATACGACACAGGCCA
	Forward: GTCAGCGCAATATAACACAG
	Reverse: GTGCAGGGTCCGAGGT
miRNA-155	RT: CCTGTTGTCTCCAGCCACAAAAGAGCACAATATTTCAGGAGACAACAGGACCCCTA
	Forward: CGCCGTTAATGCTAATCGTGA
	Reverse: CAGCCACAAAAGAGCACAAT
miRNA-218	RT: CTCAACTGGTGTCGTGGAGTCGGCAATTCAGTTGAGAGCTATGC
	Forward: CGAGTGCATTTGTGCTTGATCTA
	Reverse: TGGTGTCGTGGAGTCG
miRNA-503	RT: GTCGTATCCAGTGCGTGTCGTGGAGTCGGCAATTGCACTGGATACGACCTGCAGT
	Forward: GCGTAGCAGCGGGAACAGT
	Reverse: CCAGTGCGTGTCGTGGAGT
miRNA-16	RT: CCTGTTGTCTCCAGCCACAAAAGAGCACAATATTTCAGGAGACAACAGGCGCCAAT
	Forward: CTCGCTTCGGCAGCACA
	Reverse: AACGCTTCACGAATTTGCGT
miRNA-1228	RT: GTCGTATCCAGTGCGTGTCGTGGAGTCGGCAATTGCACTGGATACGACGGGGGG
	Forward: CGGCGTCACACCTGCCTCG
	Reverse: CAGTGCGTGTCGTGGAGTC

**Note:** miRNA, microRNA; RT, reverse transcription; Ref. gene, references gene.

### 2.5 Quantitative real-time PCR (qRT-PCR)

qRT-PCR was performed using the miRCURY LNA SYBR Green PCR Kit (Qiagen, Germany) and the LightCycler96 real-time PCR product detection system (Roche, Switzerland). The volume of the reaction mixture was 10 μl. The amplification program included the following steps: polymerase activation at 95 °C - 2 min, 50 cycles - denaturation at 95 °C - 10 s, primer annealing at 56 °C - 60 s. For the detection of reference gene (housekeeping gene) and target miRNAs, commercial kits of primers and probes miRCURY LNA miRNA PCR Assay (Qiagen, Germany) were used. The sequence of all primers used in the present study is provided in Table 3. To quantify gene expression, the 2-ΔΔCt method was used, based on the fact that the difference in the value of the “threshold cycle” (ΔCt) between the gene under study and the control gene is proportional to the level of relative expression of the gene under study.

### 2.6 Reference gene selection

The selection of reference genes was based on literature data. Since no universal genes have been found to normalize the data, the relative quantification of changes in the expression of exosomal miRNAs in biological fluids has faced a serious problem. RNU6A(U6) or SNORD44 are often used as such genes, however, they belong to the class of small nuclear RNAs with properties different from miRNAs. A number of authors recommended using hsa-miRNA-16 and hsa-miRNA-1228 as housekeeping genes to study the level of expression of circulating miRNAs in plasma or serum [[13], [14], [15], [16]]. We followed these guidelines because hsa-miRNA-16 and hsa-miRNA-1228 were described as stable and highly expressed in plasma, and we normalized to the mean of these two miRNAs. The sequence of all primers used in the present study is provided in Table 3.

### 2.7 Statistical analysis

The Student T-test, analysis of variance, the chi-square test, or the Mann-Whitney *U* test was applied, as appropriate. A probability of P < 0.05 or P < 0.001 was considered to indicate statistical significance. The statistical analyses were performed using SPSS, version 22.0, software (IBM Corp., Armonk, New York, USA), and the graphs were generated using GraphPad Prism, version 7.0 (GraphPad, San Diego, California, USA).

## 3 Results

### 3.1 Expression levels of candidate miRNAs

**Fig. 1:**
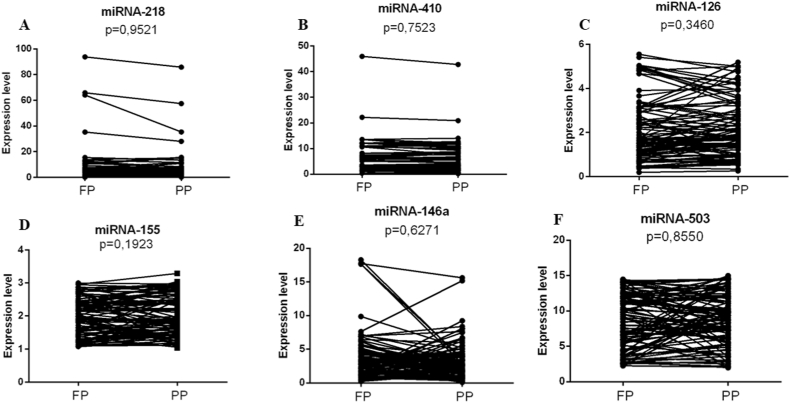
Analysis of exosomal miRNAs expression in hemorrhagic fever with renal syndrome (HFRS) patients (A–F). FP, febrile period of the disease; PP, polyuric period of the disease. The significance level (p-value) is determined using the Wilcoxon T-test.

At the first stage of the study, we conducted a pairwise comparison of plasma miRNA-146a, miRNA-126, miRNA-218, miRNA-410, miRNA-503, and miRNA-155 expression levels in patients with HFRS at different stages of the course of the disease, namely, at the stage of fever, polyuric stage HFRS and stages of convalescence. The differences were not statistically significant (P > 0.05) (Fig. 1A–F).

### 3.2 Expression levels of exosomal miRNAs

At the next stage, we compared the expression of exosomal miRNAs in patients with HFRS in groups with different course of the disease - moderate, severe, severe with complications. When comparing the expression levels of exosomal miRNAs in groups of patients with different severity of the disease, no statistically significant changes in the expression levels of miRNA-126, miRNA-218, miRNA-410, and miRNA-503 (P > 0.05) were found. Analysis of the expression of exosomal miRNA-126, miRNA-218, miRNA-410, and miRNA-503 and in the group of patients with HFRS compared with the healthy control group also did not reveal statistically significant results (P > 0.05).

### 3.3 Expression levels of exosomal miRNA-146a and miRNA-155

**Fig. 2:**
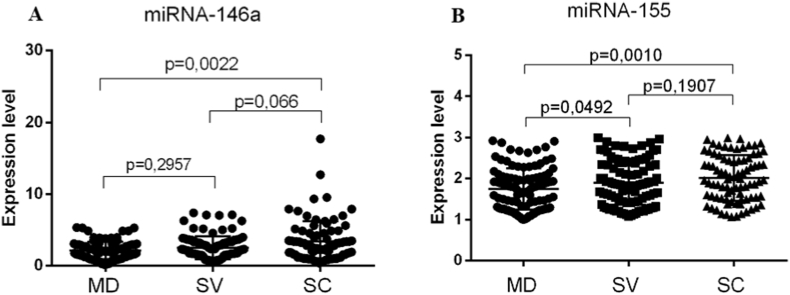
Analysis of the expression of exosomal miRNAs in patients with hemorrhagic fever with renal syndrome (HFRS) patients in groups with different forms of disease severity (A–B). (A) It has been shown that HFRS patients with severe form disease and complications have an increased expression level of exosomal miRNA-146a compared with patients with a moderate form of the disease. (B) The expression levels of miRNA-155 increased in HFRS patients with a severe form of the disease and in a severe form with complications. The significance level (p-value) was determined using the Mann-Whitney *U* test. MD, moderate form of the disease; SV, severe form of the disease; SC, severe form of the disease with complications.

**Fig. 3:**
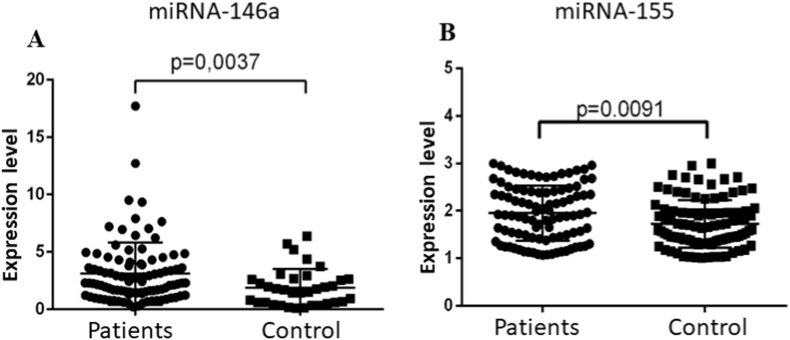
Analysis of the expression of exosomal miRNA-146a and miRNA-155 in a group of patients with hemorrhagic fever with renal syndrome (HFRS) and a healthy (control) individuals (A–B). A statistically significant increase in the expression of exosomal miRNA-146a and miRNA-155 was demonstrated in patients with HFRS compared with healthy individuals. The significance level (p-value) was determined using the Mann-Whitney *U* test.

When comparing the expression levels of exosomal miRNAs in groups of patients with different severity of the disease, a statistically significant increase in the expression level of miRNA-146a was found in patients with severe HFRS and complications (Fold change 2.694; p = 0.0022) compared with the group with moderate form of the disease (Fig. 2A). Analysis of miRNA-155 expression level demonstrated an increase in its expression both in patients with severe disease and in patients with severe HFRS and complications compared with the moderate form (Fold change 1.861; p = 0.0492; Fold change 1.976; p = 0.001, respectively) (Fig. 2B). Analysis of the expression of exosomal miRNA-155 and miRNA-146a in the group of patients with HFRS compared with the healthy control group revealed a statistically significant increase in the expression of exosomal miRNA-146a in patients with HFRS compared with healthy individuals (p < 0.05, Fig. 3A and B). Thus, our study shows that miRNA-155 and miRNA-146a can be promising markers that can predict the severe course of the disease and the development of complications in HFRS.

## 4 Discussion

In recent years, several studies have shown that miRNAs may play an important role in human diseases caused by various viruses. It is suggested that they may influence viral replication and changes in the host transcriptome during viral infection. Disturbances in miRNA expression, in turn, can alter the antiviral response. Some miRNAs are encoded by viruses, expressed in the host genome, and may contribute to the regulation of both host and viral gene expression during infection. For this reason, the relationship between virus and miRNA is a complex mechanism. Recent studies have shown that host miRNAs alter viral pathogenesis by regulating gene expression, as well as virus translation and replication [17]. The role of miRNAs in inflammatory processes and the integrity of the EC barrier has also been established [[18], [19], [20], [21]]. However, a small number of studies have been carried out on the level of miRNA expression in ECs during infection with orthohantaviruses.

Our study revealed differential expression of exosomal miRNA-155 and miRNA-146a in patients with HFRS compared with controls and in severe disease, which is confirmed by some published data. Thus, the results of the study by Chen et al. showed that live Hantavirus can induce the expression of miRNA-146a, nuclear factor kappa-B (NF-κB), and a number of pro-inflammatory cytokines in human umbilical vein endothelial cells (HUVECs) [6]. The miRNA-146a promoter has been shown to have two binding sites to the 3′-UTR of NF-kB mRNA, and NF-kB-dependent expression of miRNA-146a can be considered as a regulator of the innate immune response [22]. It is well known that miRNA-146a is involved in innate immunity and inflammatory responses in some viral infections [23]. Chen et al. concluded that NF-kB-dependent induction of miRNA-146a expression also occurs during Hantavirus, since the proteins of this virus, nucleoprotein/glycoprotein (NP/GP), contribute to the promoter activity of miRNA-146a and NF-kB expression [6]. The authors also demonstrated that miRNA-146a suppresses the expression of interferon-β (IFN-β) and promotes Hantavirus replication at the stages of the experiment using miRNA-146a mimic and anti-miRNA-146a, which may be the mechanism by which the virus avoided the host's natural immune response. Interferons (IFNs) can provide the first line of defense against viral infections, and activated NF-kB can directly determine the early production of IFN-b after virus infection [24]. Meanwhile, miR-146a has been shown to regulate type I interferon (IFN–I) activity through negative feedback through NF-kB [25]. This mechanism is considered universal, as many viruses can replicate by inhibiting IFNs synthesis in host cells. In this regard, it was also shown that high concentrations of Hantavirus inhibit the synthesis and secretion of IFN [26]. Another interesting observation that during Hantavirus, the expression of interleukin-8 (IL-8), chemokine ligand 5 (CCL5) and interferon-gamma-induced-protein-10 (IP-10) increased in the HUVECs, whose expression, in turn, was suppressed by miRNA-146a mimic [6]. That is, miRNA-146a acts as an inhibitor, attenuating the inflammatory response in an NF-kB-dependent manner. The NF-kB subunits act as a transcription factor for the expression of IL-8, CCL5 and IP-10. Accordingly, it is assumed that Hantavirus-induced expression of miRNA-146a forms an anti-inflammatory environment, providing conditions for the persistence of the virus in the host organism. Shin et al. showed an expression correlation between CCL5, IFN-b, and miRNA −146a in HUVECs infected with Hantavirus [27]. Increased miRNA −146a-5p expression led to a decrease in IFN-b expression in this cell line. MiRNA −146a probably remains one of the most studied miRNAs. It is known to inhibit signal transduction pathways leading to NF-κB activation, and NF-κB dysregulation is associated with diseases such as viral infections [28]. We also showed a statistically significant increase in the expression of miRNA-155 in patients compared with healthy volunteers and in groups with a severe course of the disease, as well as in severe cases and complications.

MiRNA-155 is induced by Toll-like receptor (TLR) family (TLR-2, TLR-3, TLR-4, and TLR-9) signaling in many cell types and generally functions as a pro-inflammatory miRNA by targeting factors that negatively regulate inflammation [[29], [30], [31]]. MiRNA-155 can promote inflammation as noted above, but in some circumstances it can also negatively regulate NF-κB activation, thereby controlling the inflammatory process [32,33]. Another process that is tightly controlled by miRNAs is the production of antibodies with a high degree of affinity. During infection/vaccination, B cells, follicular T helpers, and dendritic cells form germinal centers in lymphoid tissue. MiRNAs are an integral part of this antibody affinity maturation process, as evidenced by studies showing that germinal center formation does not occur if a global decrease in miRNA expression in B cells prevents germinal center formation. In these germinal centers, activated B cells undergo clonal expansion and mutate into their B cell receptors (somatic hypermutation), which are then tested for antigen bound by follicular dendritic cells in the presence of T follicular helper cells. B cells enter apoptosis unless their B cell receptor binds strongly to antigen, in which case they receive cell survival signals from follicular T helpers, which causes miRNA-155 to be activated, which then acts on pro-apoptotic factors such as jumonji, AT rich interactive domain 2 (JARID2) and reverses the apoptotic pathway [34]. Mutation of the B-cell receptor (through somatic hypermutation) and switching of immunoglobulin classes are regulated by miRNA. Activation-induced cytidine deaminase (AID) catalyzes mutation of the immunoglobulin locus and is an integral part of class switching and affinity maturation, but must be tightly controlled to prevent non-target mutations and excessive mutation rates that can lead to oncogenic mutations and, for unclear reasons, low-affinity and autoreactive antibodies. For tight regulation of AID expression, B-cell receptor signaling induces miRNA-155 concurrently with AID [29]. Co-induction of AID and miRNA-155 following B-cell receptor stimulation creates a system in which AID is rapidly induced and prevents immune pathology. Given the key role of miRNA-155 in the germinal center response, dysregulation of miRNA-155 could lead to immune dysfunction in humans. Supporting this is that B cells of older people have a higher level of miRNA-155 expression compared to younger people, and this prevents class switching in B cells of older people due to increased downregulation of AID. These data indicate that modulation of miRNA immunity is a delicately balanced process, and increased susceptibility to infection and possibly poor response to vaccines in the elderly may be due in part to age-related miRNA dysregulation [35,36]. Hantavirus infection upregulates miRNA-155 expression in HUVECs, which, in addition to the inflammatory response, has recently been shown to play a role in the regulation of angiogenesis or vascular integrity [37]. MiRNA-155 is reported to regulate adhesive junction disassembly, cell migration, and cell morphology, which contribute to changes in vascular permeability. It is known that miRNA-155 is critical for cell-mediated immune responses and is expressed in B cells, T cells, and macrophages [38]. Expression of miRNA-155 is associated with pro-inflammatory transcription and is induced in response to inflammatory stimuli within a few hours. Moreover, miRNA-155 regulates macrophage responses by modulating cytokine production [39]. Cytokine signaling suppressor 1 (SOCS1) is one of the major negative regulators of the Janus kinase 2 (JAK)/signal transducer and activator of transcription (STAT) pathway and mediates the inhibition of pro-inflammatory cytokines including tumor necrosis factor-α (TNF-α), interleukin-6 (IL-6), and interferon-γ (IFN- γ). MiRNA-155 promotes the production of these cytokines by downregulating SOCS1 and significantly modulates the inflammatory response. More recently, miRNA-155 antagonists have been found to decrease TNF-α and IL-6 production and increase anti-inflammatory cytokine levels by upregulating SOCS1 expression [40,41].

MiRNA-155, whose expression level in our study differed significantly in patients with different disease severity, has a number of known biological functions, including induction of Toll-like receptor (TLR) activation in monocytes/macrophages and modulation of TLR signaling, promoting pro-inflammatory cellular responses and initiating systemic inflammatory responses [42]. A single miRNA has been shown to influence the expression of hundreds of target genes. However, any effect of a single target on its function is unclear. According to precise theories, the function of a single miRNA-mRNA interaction varies depending on cell type and biological pattern. The decisive role of miRNA-155-mediated regulation of SOCS1 in specific cellular and biological mechanisms does not exclude the possible involvement of other targets of miRNA-155 [43]. Studies in larger patient cohorts are needed to understand the impact of miRNA-155 and SOCS1 interaction on the progression of viral infections. These studies will provide knowledge in terms of pathophysiology of diseases and research into therapeutic target molecules.

This study did not reveal any statistically significant differences in the expression levels of miRNA-126, miRNA-218, miRNA-410, and miRNA-503. It is known that inflammatory stimuli, such as IFNs, lysophosphatidic acid (LPA), interleukins, TNF, and ligands of TLR, which are induced during viral infection, change miRNA expression in ECs, including miRNA-126, which is involved in the inflammatory response, angiogenesis, and vascular integrity [[17], [18], [19], [20],[44], [45], [46]]. Reduced expression of a number of miRNAs, including miRNA-126, was observed in patients with Crimean-Congo hemorrhagic fever (CCHF) compared with the control group, while the expression of miRNA-126-3p decreased by 45 times, which is associated with the involvement of miRNA-126-3p in the production of interferon I type (IFN–I) and modulation of the pathophysiology of the immune response in patients [47,48]. A study describing the expression profile of miRNAs in liver tissues for the first time shows increased expression of miRNA-126, which activates the janus kinase 1 (JAK1)/signal transducer and activator of transcription 3 (STAT3) pathway by inhibiting zinc finger E-box binding homeobox 1 (ZEB1) in dengue fever *in vivo* [49].

A recent study showed that miRNA-126-3p expression during the febrile and convalescent phases in children infected with dengue virus was significantly lower than in children with acute fever not infected with dengue virus, suggesting that miRNA-126-3p is involved in the pathogenesis of the dengue virus [50]. Infection of ECs with Andes orthohantavirus (ANDV) led to a decrease in miRNA expression in endothelium, including miRNA-218, which is associated with the regulation of ECs migration and vascular permeability, resulting in disintegration of intercellular interactions and changes in vascular permeability [37,[51], [52], [53], [54]]. This suggests that in virus-infected ECs, reduced expression of miR-218 enhances vascular endothelial growth factor (VEGF)-targeted permeability by upregulating roundabout guidance receptor 1 (Robo1) and downregulating roundabout guidance receptor 4 (Robo4), which stabilizes the vasculature by counteracting VEGF signaling responses that lead to increased endothelial vascular permeability [55,56]. Changes in the expression of miRNA-126, which regulates the integrity of blood vessels by suppressing the expression of mRNA of the sprouty related EVH1 domain containing 1 (SPRED1) and phosphoinositide-3-kinase regulatory subunit 2 (PIK3R2), led to an increase in the expression of these genes [37,57,58]. In accordance with the increased expression of SPRED1, the level of phosphocophilin was reduced in ECs infected with Hantavirus, which increased the disruption of intercellular interaction [37]. Another study revealed significant changes in the expression of 14 miRNAs, including miRNA-218, in dengue and influenza viruses, indicating that these miRNAs are regulators of pathogenesis in viral infections [59].

There is information about the relationship between reduced expression of miRNA-503 and activation of cofilin, which reduces the stability of adhesive junctions in ECs infected with Hantavirus [37]. The infection resulted in a 77-fold decrease in miR-503 expression in ECs, where miRNA-503 regulates the expression of cyclin D1 [60]. While a decrease in cyclin D1 expression demonstrates increased cell adhesion, an increase in cyclin D1 expression reduces cell adhesion and enhances cell migration by inhibiting Ras homolog family member A (RhoA)/Rho‐kinase (ROCK) response signals. LIM kinase (LIMK), which phosphorylates cofilin, is a key substrate for ROCK, and thus downregulation of miRNA-503 may also contribute to increased cofilin activity and increased endothelium permeability seen in Hantavirus infection.

According to the results of microarray methods in HUVECs infected with Hantavirus, a sharp 3400-fold decrease in miRNA-410 expression was demonstrated [37]. Aberrant expression of miRNA-410 has been identified in a variety of diseases, including cancer, inflammation, and autoimmune diseases, which is reflected in many biological processes such as proliferation, apoptosis, stem cell differentiation, and drug resistance [61,62]. It is currently unclear how such variability in miRNA-410 expression in Hantavirus-infected ECs could play a role in the regulation of cell activation or immune recognition. However, the dramatic decrease in miRNA-410 expression suggests that downregulation of this miRNA may play an important role in Hantavirus infection within their unique endothelial niche.

Despite the fact that we did not show statistically significant differences in the expression of miRNA-126, miRNA-218, miRNA-410 and miRNA-503, neither depending on the period of the disease, nor depending on the severity of its course, some studies have described their important role in Hantavirus infection.

## 5 Conclusion

In this study we revealed that exosomal miRNA-155 and miRNA-146a are associated with severe HFRS. The fact that these microRNAs can indeed participate in the pathogenesis of HFRS is also confirmed by the fact that they can act on various immune cells, making recipient cells more susceptible to viral infection. However, further research is needed in this direction. Understanding the role of miRNAs in the pathogenesis of infections caused by Orthohantaviruses is of great interest, since the identification of new biomarkers and the development of miRNA-based therapeutics for the treatment of viral infections, including HFRS, is a promising direction.

## Ethics approval and consent to participate

The approval was provided by the ethics committee of Ufa Federal Research Center of the Russian Academy of Sciences (Ufa, Russia) and Republican Clinical Infectious Diseases Hospital (Ufa, Russia).

## Human and animal rights

No animals were used for studies that are base of this research. All the human's procedure was followed in accordance with the ethical standards of the committee responsible for human experimentation (institutional and national), and with the Helsinki Declaration of 1975, as revised in 2013 (http://ethics.iit.edu/ecodes/node/3931).

## Consent for publication

Written informed consent was obtained from all individual participants included in the study.

## Author contributions

Investigation, Writing - original draft and Resources: Irina Gilyazova; Writing - review and editing, Conceptualization and Project administration: Ilgiz Gareev and Ozal Beylerli; Formal analysis and Methodology: Elizaveta Ivanova and Dilara Asadullina; Data curation: Guzel Khasanova, Adel Izmailov and Aliya Khasanova; Validation and Visualization: Gulshat Gilyazova, Elza Khusnutdinova and Guoqing Wang; Funding acquisition and Supervision: Valentin Pavlov and Elza Khusnutdinova. All authors have read and agreed to the published version of the manuscript.

## Funding

The study was financially supported by 10.13039/501100002261Russian Foundation for Basic Research (RFBR) and 10.13039/501100001809National Natural Science Foundation of China (NSFC) in the framework of scientific project № 21-515- 53017 NSFC_a. This work was supported by the Bashkir State Medical University Strategic Academic Leadership Program (PRIORITY-2030).

## Declaration of competing interest

The authors declare no conflict of interest, financial or otherwise.
